# Exploring the Nature of Authority Over, and Ownership of Data Generated by Archaeological Lidar Projects in Latin America

**DOI:** 10.1007/s11759-022-09464-z

**Published:** 2022-11-19

**Authors:** Anna S. Cohen, Juan Carlos Fernandez-Diaz, Amanda Meeks

**Affiliations:** 1 Department of Sociology and Anthropology, Utah State University, Old Main 0730, Logan, UT 84322 USA; 2 National Center for Airborne Laser Mapping, University of Houston, 5000 Gulf Freeway, Houston, TX 77204 USA

**Keywords:** Lidar, Ownership, Authoritative voice, Equity, Latin America

## Abstract

Data ownership and accessibility are critical issues across academia, but especially in fields that touch upon digital heritage that relates to pre-colonial/colonial societies. Who can access spatial datasets about pre-colonial landscapes, who is writing about these topics, and who, by extension, is considered an authority on these topics? This paper explores data ownership, gender, and local affiliation by examining publications on archaeological lidar in Latin America between 2011 and 2021. For over 10 years, archaeological remote sensing derived from lidar has transformed research in Latin America and especially in Mesoamerica, yet there are numerous issues related to data ownership and authoritative voice that remain unresolved. This study shows that publication authorship, including first and co-authorship, is dominated by male researchers at US institutions while women and individuals associated with institutions in Latin America are poorly represented. The limited representation of authors with local or community affiliations suggests that local authoritative voices are largely muted in archaeological lidar research in the region. We discuss working toward more collaborative lidar research in Latin America.

## Résumé

La propriété des données et leur accessibilité sont des questions critiques à travers la recherche universitaire, mais plus particulièrement dans les secteurs ayant trait au patrimoine numérique en lien avec les sociétés pré-coloniales/coloniales. Qui peut accéder aux ensembles de données spatiales relatifs aux paysages pré-coloniaux, qui écrit sur ces thèmes et qui par extension est considéré comme ayant autorité sur ces derniers ? Cet article est une étude de la propriété des données, du genre et de l'affiliation locale par l'examen de publications sur le lidar (*light detection and ranging*) archéologique en Amérique Latine entre 2011 et 2021. Pendant plus de dix ans, la télédétection archéologique ayant découlé de lidar a transformé la recherche en Amérique Latine, en particulier en Mésoamérique. Pourtant, de nombreux problèmes demeurent sans solution relativement à la propriété des données et à la voix faisant autorité. Cette étude démontre que la paternité de la publication, y compris la première et la co-paternité, est dominée par les chercheurs mâles dans les institutions nord-américaines alors que les femmes et les individus associés à des institutions en Amérique Latine ne sont que fort peu représentés. La représentation limitée des auteurs ayant des affiliations locales ou communautaires suggère que les voix locales faisant autorité sont largement réprimées au sein de la recherche lidar archéologique dans la région. Nous engageons une discussion sur un travail en faveur d'une recherche lidar plus collaborative en Amérique Latine.

## Resumen

La propiedad y la accesibilidad de los datos son cuestiones críticas en todo el mundo académico, pero especialmente en los campos que abordan el patrimonio digital relacionado con las sociedades precoloniales/coloniales. ¿Quiénes pueden acceder a conjuntos de datos espaciales sobre paisajes precoloniales, quiénes escriben sobre estos temas y quiénes, por lo tanto, son considerados autoridades en estos temas? Este documento explora la propiedad de los datos, el género y la afiliación local mediante el examen de publicaciones sobre la Detección de luz y rango (LiDAR, por sus siglas en inglés) arqueológica en América Latina entre 2011 y 2021. Durante más de diez años, la teledetección arqueológica derivada de LiDAR ha transformado la investigación en América Latina y especialmente en Mesoamérica; sin embargo, hay numerosos problemas relacionados con la propiedad de los datos y las voces autorizadas que siguen sin resolverse. Este estudio muestra que la autoría de publicaciones, incluidas la primera autoría y la coautoría, está dominada por investigadores masculinos en instituciones estadounidenses, mientras que las mujeres y las personas asociadas con instituciones en América Latina están poco representadas. La representación limitada de autores con afiliaciones locales o comunitarias sugiere que las voces autorizadas locales se silencian en gran medida en la investigación arqueológica LiDAR en la región. Abordamos la cuestión de trabajar hacia una investigación LiDAR más colaborativa en América Latina.

## Introduction

The questions of who owns or controls data, who can access them, and how they are represented are critical issues across academia, but especially in fields that touch upon digital heritage that relates to pre-colonial/colonial societies. For example, who can access spatial datasets about pre-colonial landscapes in Latin America, who is writing about these topics, and who, by extension, is considered an authority on these topics? Are the authors from the countries and communities where data collection occurred? In recent discussions of data accessibility and data management, studies have focused on best practices, cautionary tales, and conceptual theses about “big data” as they relate to remote sensing, radiocarbon determinations, and other enormous databases that archaeologists harness in their research (Bevan, 2015; Cohen et al., 2020; Fernandez-Diaz et al., 2018; Gattiglia, 2015; McCoy, 2017; VanValkenburgh & Dufton, 2020). Other papers focus on diversity, equity, and inclusion in archaeological research, especially as these relate to gender and intersectionality (eg., Goldstein et al., 2018; Hoggarth et al., 2021). One method of assessing diversity in archaeological research uses the names of article authors or grant recipients to assign gender identity, an approach which is useful, but which has been critiqued for its lack of intersectionality or use in multi-issue studies (Heath-Stout, 2020). Missing from many of these archaeological studies about data access and management is any detailed discussion about authoritative voice and writing, and how in academia authorship facilitates ownership over the data and narrative presented in a published text (Jeppson, 1997; Nakata, 2018).

In this paper, we explore data ownership, access, and diversity (gender and author affiliation) by presenting a study that examines the authorship of publications on archaeological lidar use in Latin America. In doing so, this paper takes an academic-centric approach to data ownership: we contend that publications are a way for authors to claim authority over ideas, related digital datasets, and narratives, which can have the unintended effect of silencing the voices of others, particularly local community members (Jeppson, 1997). While other forms of data ownership do exist and data are created outside of academic systems, authorship is the *primary* means by which scholars demonstrate their authoritative voice and data use within an academic environment. As emphasized vis-à-vis the data publishing model, publishing is a claim to authority on subject matter (see also Bennett et al., 2022; Reddix-Smalls, 2014; Sanger & Barnett, 2021). One such data medium is airborne lidar or airborne laser scanning [ALS], which involves the collection of remote datasets, and which has substantially influenced archaeological projects and theoretical arguments in recent years. In heavily vegetated places such as Mesoamerica, the ability to filter out data that are undesirable for archaeological purposes, for example, lidar returns from vegetation and modern infrastructure, has enabled researchers to document and visualize human-modified landscapes at a scale that was impossible previously.

ALS data collection requires significant upfront monetary resources, and because of this, until recently, there has been a limited number of academic research projects that can afford to collect, analyze, and store such datasets. Now that lidar scans are used more widely in the archaeology of Latin America, it is an opportune time to examine which projects and individuals publish these scans and the resulting interpretations. In doing so, we assess the state of the field in archaeological lidar via authorship (ie., a proxy measure of academic authority) and we can provide suggestions for more equitable research collaborations, especially in terms of co-authors and their relationship to the local and Indigenous community members where scanning occurred. A more equitable and balanced research publication should include proportional numbers of male and female authors that reflect the general population, and at the least, equal numbers of co-authors with affiliations in Latin America.

Here, we show that publication authorship, including leading and co-authorship, is dominated by senior male researchers at US institutions. Women and especially individuals associated with institutions in Latin America are poorly represented among studies that appeared in print between January 2011, the year that the first major paper on archaeological lidar in the region was published, and October 2021, preceding the submission deadline for this special issue. With a growing number of publications on the topic and the potential for increasing access to lidar datasets, there may be an emerging trend toward increasing gender balance. It is unclear whether local affiliation is also moving in this direction. Representation of Indigenous communities in authorship (and by extension, voice) is nonexistent in our sample. After discussing our dataset, we highlight a few suggestions for moving toward more inclusive and balanced digital ownership. Although there are a growing number of research projects led and supported by Indigenous academics and community members in the US and Canada (eg., Cipolla et al., 2019; Colwell, 2016; Gonzalez, 2016; Marek-Martinez, 2021; Silliman, 2008), the situation in other countries is different and, as González-Ruibal (2019) warns, the experiences of both settler colonialism and indigeneity are specific and not necessarily transferable, which we discuss in a later section. To be clear, this is not intended as a critique of recent lidar projects; rather, we view this paper as an opportunity to consider issues such as authorship and authoritative voice, and what “counts” as data access and ownership in digital archaeological projects.

## Meta-analyses and Archaeological Lidar

Generally, archaeological remote sensing and the digitization of archaeology have facilitated collaboration and data sharing between scholars, government entities, and the broader public, but there are numerous issues related to data accessibility and storage, as well as the integration of Indigenous or descendant communities in these projects, that remain unresolved. In discussions of digital heritage and “big archaeology,” scholars have focused on how we constitute digital spatial data (Bevan, 2015; Gupta et al., 2020), open access frameworks and datasets (Dallas, 2015; Fredheim, 2020; Huggett, 2012; Kansa et al., 2020; Opitz, 2016), and the possibilities of working within “citizen science” contexts (Álvarez Larrain & McCall, 2019; Forest et al., 2020; Lambers et al., 2019; Stewart et al., 2020) (see also VanValkenburgh & Dufton, 2020). Recent scholarship on digital heritage highlights how digital approaches can facilitate collaboration with local stakeholders, such as Indigenous and descendant communities (Hill & DeHass, 2018), with most work focusing on museum contexts (Brown & Nicholas, 2012; Dawson et al., 2011; Hollinger et al., 2013). As Gupta et al. (2020) point out, related discussions concentrated on data governance and maintaining spatial databases do not explicitly outline the legal and policy environment in which archaeologists use these databases, especially when it comes to Indigenous peoples (see also Reddix-Smalls, 2014; Sanger & Barnett, 2021).

Since the early 2000s, the remote collection of lidar datasets for archaeological purposes has increasingly become a major tool within archaeology. This coincides with the so-called “geospatial revolution” (Chase et al., 2012) that is visible via an uptick in publishing on geospatial and remote sensing topics since 2005 (McCoy, 2021). Although applied earlier in European contexts (Bewley et al., 2005; Shell & Roughley, 2004), since 2009 in Latin America, over 40 archaeological projects have sought to integrate these datasets into their research programs. Long used for environmental and other geopolitical purposes, lidar datasets can provide an environmental record of a given landscape at the time of scanning, but they are also capable of documenting cultural features hidden beneath vegetation, which serves a critical role in these times of diminishing Indigenous landscapes (McSweeney et al., 2014). From an anthropological perspective, the collection of lidar data has in many ways fundamentally altered how scholars document, visualize, and interpret pre-colonial landscapes. In Latin America, this has included modified survey methods using lidar-derived visualizations, 3D and other models of features, and landscape-wide conceptualizations of topics like urbanization and monumentality (eg., Canuto et al., 2018; Chase et al., 2012; Ebert et al., 2016; Fisher et al., 2017; Inomata et al., 2020; Khan et al., 2017; Rosenswig et al., 2013; Sugiyama et al., 2021; Stenborg et al., 2018; VanValkenburgh et al., 2020; Venter et al., 2018; Yaeger et al., 2016).

In many ways, the collection, analysis, and use of lidar data create distinct data governance challenges compared to those associated with other types of remote sensing technologies. In most cases, lidar and other drone-acquired imagery involves data collection over large areas, generally without government or local stakeholder involvement (Fernandez-Diaz & Cohen, 2020). This is different from existing remote datasets, such as aerial photographs, satellites, or even legacy lidar data, all of which have their own accessibility issues, but which often do have a legal framework within which the spatial data are collected or shared (Fernandez-Diaz & Cohen, 2020; McLeester & Casana, 2021). In contrast, the situation for future lidar scans is murkier: between 2002 and (November) 2020 (when the US government left the Open Skies Treaty), US-sponsored lidar collections in Latin America arguably should be subject to the principles set forth in the Open Skies Treaty, an agreement between 30+ state parties, which used to include the US, Canada, and all European countries, that stipulates that the observed countries must receive a copy of all data collected from a flight (Arms Control Association, 2019). This means that if US researchers used federal (eg., National Science Foundation) funds to collect remote datasets in Latin American countries, they should provide copies of all material to the host country. Since the US withdrew from the Treaty in 2020, it is unclear whether future lidar scans will be subject to international legal policies.

This lack of clarity on legal frameworks and lidar scanning means that meta-archaeological analyses are particularly important for assessing archaeological lidar data collection, access, and publication. Broadly situated in earlier literature on the socio-politics and philosophy of archaeological practice (Conkey & Spector, 1984; Gero, 1985; Trigger, 2006; Wylie, 2002), meta-archaeological analyses have examined, for example, who is awarded major research funding (Goldstein et al., 2018), sexual and other forms of harassment within the field (Clancy et al., 2014; Meyers et al., 2018; Voss, 2021a, 2021b), who is publishing in archaeology (Bardolph, 2014; Heath-Stout, 2020; Tushingham et al., 2017), and where they are publishing (Beck et al., 2021). Within geospatial archaeology, studies have assessed the number of overall publications in terms of geographical origin and topic (Agapiou & Lysandrou, 2015; McCoy, 2021), and ethical considerations in remote sensing (Cohen et al., 2020; Davis & Sanger, 2021; Fernandez-Diaz et al., 2018; Kersel & Hill, 2019). In general, these studies support the observation that geospatial tools and publications are most associated with wealthy countries and universities, especially in European contexts (see also Bevan, 2015; Opitz & Herrmann, 2018).

In this meta-archaeological study, we view publications as constituting predominant authoritative voice and thus a type of ownership over digital heritage. Publications are fundamental for circulating research among academic and other institutions, they are expected by most funding agencies, and they are recognized for academic promotion. Important for our discussion below, publications are a way for authors to claim authority over ideas and related datasets.

## Producing, Accessing, and Publishing an Archaeological Lidar Dataset

Before turning to publications, it is relevant to briefly outline how archaeologists collect ALS data, and the data access policies that are in place for these datasets. Here, we mostly draw upon the experience of the second author (Fernandez-Diaz) who has personally collected over 30 archaeological datasets in Mesoamerica between 2009 and 2020 (eg., Canuto et al., 2018; Fisher et al., 2017; Inomata et al., 2020; Sugiyama et al., 2021). These datasets are archived in three separate locations: at the National Center for Airborne Laser Mapping (NCALM) at the University of Houston, with the researchers themselves, and in some cases with the observed country; collections sponsored by the US National Science Foundation (NSF) are also archived through Open Topography (https://opentopography.org/), although data access is typically restricted due to a lack of open access guidelines in place within the observed countries (Fernandez-Diaz & Cohen, 2020). As foreign entities, US operators need to obtain government permits to conduct large-scale scans in Latin America with crewed aircrafts. We recognize that some projects not collected by NCALM may have different policies regarding smaller scans (via drones, for example) and data storage policies. Other differences in data access and publication are clear both anecdotally and in the meta-analysis of publications presented in this paper.

Similar to other types of remote data collection, the area of ALS collection is informed by extant research knowledge of the region and prevailing budgetary constraints. Based on published research methods, it is unusual for the scan region to be defined through consultation with communities living near archaeological sites who may include Indigenous or other descendant communities, and stakeholders using the land for agricultural or other activities (for an exception, see Palka et al., 2020). The NCALM is an NSF-supported center, and it collects data both for projects that are NSF-funded, but also through direct contract with research groups, agencies, and non-governmental organizations. The first step is to develop flight and logistics plans based on the project specifics (location, measurement density, research objectives). Afterward, a budget is developed, most of which involves labor, an airplane, and transporting equipment to the project location. If most of the resources are to be used toward project area scanning, with only a quarter or less for mobilization, then the total budget in Mesoamerica should be more than US $100,000, which will typically allow for data collection over more than 300 km^2^ with measurement densities of > 10 pulses/m^2^. NCALM scans typically range between 40 and 300 km^2^, though the center has collected both much smaller (~ 10 km^2^) and much larger (over 5,000 km^2^) datasets.

After field collection, the different airborne data streams (ranging, positioning, and navigation) are taken to the laboratory at the University of Houston for data processing and the production of the results (point clouds and rasters) following procedures described in Fernandez-Diaz et al. (2014). The results are then made available to researchers, who often work with lidar specialists (eg., the second author) to refine the results and create enhanced visualizations from the terrain models, 3D animations, and many other data that can also be integrated into field and laboratory contexts. Increasingly, archaeologists with access to the appropriate equipment and software can manipulate lidar point clouds and rasters without the help of specialists. While this is in some ways a positive trend toward self-sufficiency in archaeological data processing, this also means that individuals who can access expensive resources are becoming specialists over others who may be based at less affluent locations and do not have such resources. After this, there is no set publication procedure for either the ALS data or the results of archaeological interpretations. The NSF states that data should be open after an embargo period and published within a “reasonable” period (https://www.nsf.gov/bfa/dias/policy/dmp.jsp), but there is no clarity regarding what length of time is considered ‘reasonable.’ Because of the lack of clear guidance within the observed countries and fears that open data may lead to looting or destruction of cultural resources, none of the archaeological datasets funded by NSF to date are open access (Fernandez-Diaz & Cohen, 2020). Data collection funded by private entities is subject to their unique data policies; thus, their access is mostly restricted.

## Publishing About Lidar

### Methods

To assess data ownership in lidar research via publications, we narrowed our focus to two categories that can be identified through authorship: gender and university affiliation. We started by focusing on widely read archaeology journals that cover world archaeology (compare with Agapiou & Lysandrou, 2015; Bardolph, 2014; Heath-Stout, 2020; McCoy, 2021), but we also searched within high impact science journals where lidar data have been published recently, including *Science*, *Nature*, and *PLOS ONE* (Table 1). We included one other journal (MDPI *Remote Sensing*) based on personal knowledge of published lidar datasets from Latin America. Since they are difficult to track down and are in some cases not publicly available or open access, we did not include book chapters, gray literature reports, exclusive remote sensing (ie., non-archaeological) journals, or non-English publications. We searched for relevant articles within each journal in turn.

**Table 1:** Archaeology and high impact scientific journals with publications about lidar in Latin America, from January 2011 through October 2021 (last date based on submission of article)

Journal name	Number of publications	Publication year
Journal of Archaeological Science	4	2011, 2013, 2015, 2017
Proceedings of the National Academy of Sciences	3	2012, 2014, 2019
World Archaeology	2	2014, 2021
Remote Sensing	7	2014, 2017, 2020, 2021
Advances in Archaeological Practice	11	2014, 2016, 2019
PLOS One	7	2016, 2018, 2019, 2021
Latin American Antiquity	4	2016, 2019, 2021
Quaternary International	1	2017
Journal of Anthropological Archaeology	5	2017, 2019, 2021
Science	1	2018
Journal of Field Archaeology	2	2018, 2020
Antiquity	1	2018
Ancient Mesoamerica	3	2018, 2019
Nature	2	2018, 2020
Journal of Social Archaeology	1	2020
Nature Human Behavior	1	2021

Using journal search engines, we used keywords such as “lidar,” “Mesoamerica,” and/or “Latin America.” Since lidar was not used in archaeological work in Latin America until 2009, we filtered for publication years between January 2009 and October 2021 (to meet the submission deadline for this study). We were also able to cross-check our article results with the publication database at NCALM, which has been responsible for collecting an estimated 80% of the larger (> 40 km^2^) archaeological lidar datasets in Mesoamerica. There were articles that mentioned lidar research generally, or that use a lidar-derived image like a hillshade, but they do not focus specifically on lidar data collection, nor do they discuss using the datasets for analysis and interpretation. We therefore discounted these articles to gain a better understanding of who is behind the collection, funding, and analysis of lidar data.

The next step was to record first authors and co-authors into a spreadsheet for each journal article, along with the respective affiliation(s) listed for that particular paper. After listing each author’s name, we researched each author, to see what gender they might identify with through official institutional, personal or project websites, publicly available CVs and biographies, and in some cases social media postings. As discussed elsewhere (Heath-Stout, 2020), a survey in which we ask authors about gender identity would be the most meaningful for addressing intersectionality and gender inclusion. Here, we interpreted gender identity based on available information such as cultural naming conventions in Euro-American and Latin American contexts and given that the field of lidar-based archaeological research is relatively small, we also relied on our personal knowledge of many of the co-authors. While our gender assumptions are imperfect, a recent study shows that 1% or less of authors of archaeology publications identify as transgender, queer, or other (versus male or female) (Heath-Stout, 2020: Table 2) and, as such, we believe that our gender assumptions are unlikely to affect the overall gendered trends highlighted in this paper.

**Table 2:** Number of papers dealing with archaeological lidar data in Latin America by year and authors broken down into gender, foreign (ie., no professional affiliation with the country in which the scan occurred), and local affiliation/community (A/C) (ie., professional or community affiliation with the country in which the scan occurred) categories

Year	#Papers	#Male authors	#Male first authors	#Female authors	#Female first authors	#Foreign authors	# Local A/C authors	# Local A/C first authors	Total listed authors
2011	1	7	1	1	0	7	1	0	8
2012	1	4	1	1	0	4	1	0	5
2013	1	2	1	2	0	2	2	0	4
2014	7	23	5	13	2	34	2	0	36
2015	1	2	1	1	0	3	0	0	3
2016	10	31	6	16	4	36	11	0	47
2017	4	16	4	6	0	20	2	0	22
2018	7	37	6	13	1	33	17	1	50
2019	6	22	4	9	2	27	4	1	31
2020	5	14	2	10	3	18	6	0	24
2021	12	48	10	21	2	44	25	1	69
Total	55	206	41	93	14	228	71	3	299

The number of first authors associated with each of these categories is also included

University affiliation(s) were also noted as a proxy for local affiliation with the country in which data were collected. While an affiliation with, for example, a Mexican institution does not always imply that the co-author is a Mexican national or closely affiliated with local communities near the archaeological site(s) of interest, this affiliation is a step toward more meaningful representation (ie., voice) within the field than an affiliation with a US or European institution. On the other hand, we also recognize the reverse situation, where a particular individual may have an affiliation with a US or European institution but may be a native of the country where the research is performed or have a heritage connection to that country or the surrounding region. Given our familiarity with many of the authors in the field or through CV or biographical searches, we were able to account for several cases of authors with foreign affiliations but well-established heritage or local connections, such as the second author of the present study (a Honduran national with a US affiliation).

**Figure 1:**
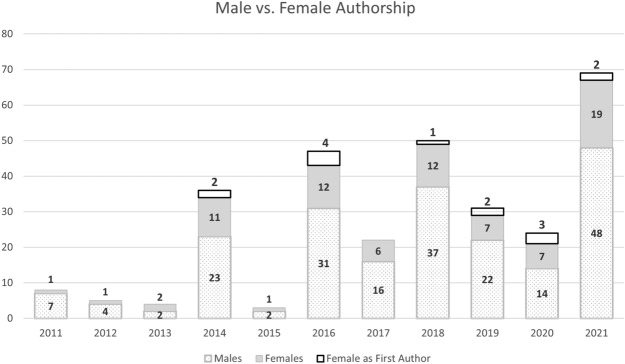
Breakdown of total authors by year according to gender. The total number of female authors is in the top two sections of each bar; the top section represents the number of females as first author

**Figure 2:**
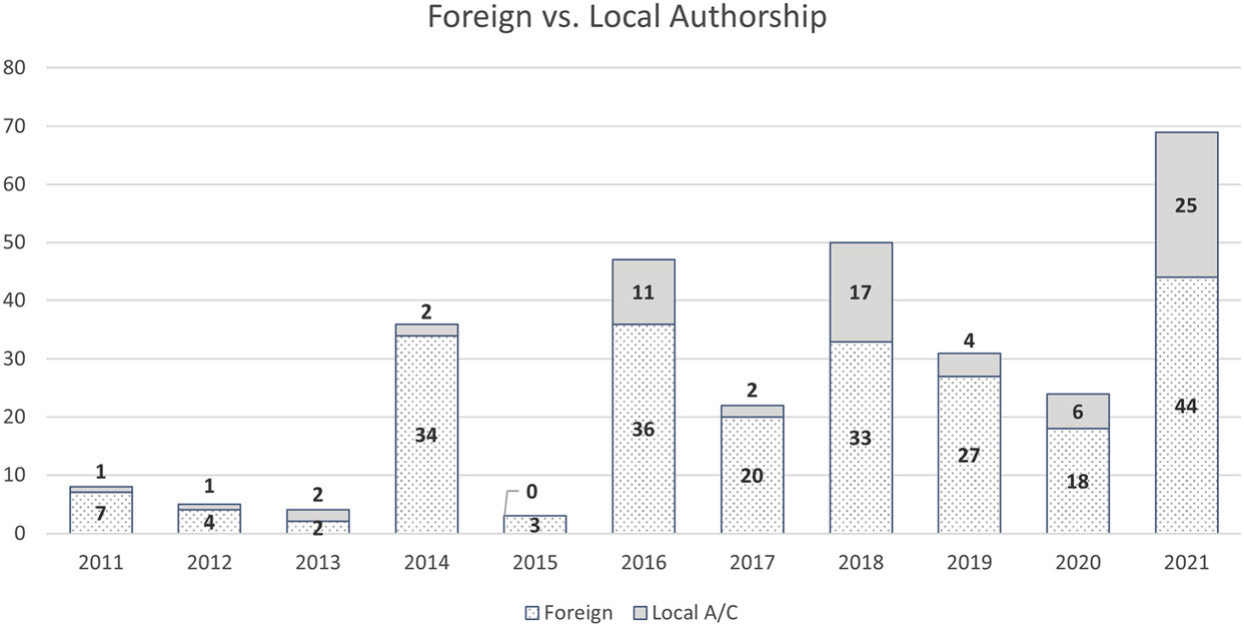
Total number of authors by year according to foreign (ie., no professional affiliation with the country in which the scan occurred) and local affiliation/community (A/C) (ie., professional or community affiliation with the country in which the scan occurred) categories

We compiled the paper and authorship information in an Excel spreadsheet where there was an individual record (row) for each author and their listed affiliation for each publication. The recorded data were standardized for consistency, making sure, for example, that the name of each individual was consistent throughout our database despite how it was listed on any given publication. We also researched each author through web and social media pages, and we confirmed gender and gathered additional information, including academic stage—eg., student, Ph.D. candidate, post doc, early career faculty, senior faculty—at time of publication, year, and institution of Ph.D. attainment, country of birth, etc. We do not report all these results here but will do so in a future publication. After the database was standardized, we used the power query options in Excel to count the number of unique individuals that have published, to obtain aggregate metrics on authorship breakdown per gender, local/foreign affiliation or connection, and year of publication. The resulting data are presented in Table 2 and in Figures 1 and 2.

## Results

A total of 16 journals with 55 papers published between January 2011 and October 2021 met our criteria for content about archaeological lidar in Latin America. As expected, over the past 10 years, there has been an increase in the number of lidar papers and thus an increase in the number of authors (Table 2). There are 299 authorship records in our database for the 55 papers that met our inclusion criteria. Of these, 206 (68.9%) are male authors and 93 (31.1%) are female (Table 2, Figure 1). Several individuals have been listed as authors on two or more publications; thus, the number of unique individuals is 198, comprised of 129 males (65.2%) and 69 females (34%). Less than a quarter of the total authorship records (71 or 23.7%) correspond to authors with a local affiliation or connection (ie., someone affiliated with a university or community in the country where the lidar data were collected) (Figure 2). In 2021, locally affiliated authorship was highest at 25 out of 69 total authors (36%). Note that in 2013, 2 of 4 total authors were locally affiliated, but since only one publication came out that year this single source sample cannot be considered representative of the overall trend.

In addition to being included as an author on a publication, the order of authors often matters in terms of both publication and data ownership. The first author is responsible for creating one narrative throughout the text, which means that they control which information is presented and the way it is presented. As such, we looked at the assumed gender and affiliation of first authors for each publication (Table 2, Figure 1). Co-author gender identity could be revealing in a different way, and we will examine this dynamic in a future study. Nearly every year, the numbers of male first authors are greater than the number of female first authors. In years with the smallest number of publications (2011, 2012, 2013, 2015, and 2017), there are no female first authors. For years with 5 to 12 publications, the number of female first authors ranges from 1 out of 7 (14%) in 2018 to 3 out of 5 (60%) in 2020. In 2021, the year with the greatest number of publications overall, 2 out of 12 (17%) of first authors are female.

The situation is more extreme in the case of first authors with local affiliations. In our sample, there were only three papers, one by an author who holds a professional affiliation with a country where scanning occurred (Punzo Díaz, 2020) and another two by authors that hold foreign affiliations but who are originally from the country in which the research was conducted (de Souza et al., 2018; Ramírez-Núñez et al., 2019). We recognize that our results may be affected by the inclusion of articles published in exclusively Spanish language journals, government reports, or the proceedings of local meetings, but these are often available only through direct request, and thus, can be more challenging to consult than English language publications. For example, one of the earlier ALS projects in Mesoamerica was led by a local female researcher; however, details of that project were published in government report proceedings and only made available on a personal website (Zetina Gutiérrez, 2013). In another case, between 2015 and 2016, the government of Ecuador undertook significant efforts to map several important archaeological zones, but this information only appears publicly in a YouTube presentation by a US-based scholar (Zeidler, 2021). As discussed below, these examples illustrate how language and funding are major barriers for local researchers in Latin America. It should come as no surprise then, that scholars who are based in Latin America are typically not well represented as first authors in English language publications or, as a direct result, owners of these spatial datasets.

## Discussion

Despite some of the limitations (eg., assumed gender) in the current dataset, we believe that the trends and metrics presented here are representative of the state of the field. These observations are reinforced by our informal and anecdotal experiences and impressions. We view this study as a means of encouraging further discussion and self-reflection within the subfield of lidar-based research in Latin America, and we see this as a first step toward a more formal and extensive meta-study regarding ownership and diversity in ALS research projects. We highlight a few areas for further discussion below.

In terms of gender and authorship, all authorship is dominated by men. Male dominance in archaeological publications has been observed in other gendered meta-analyses for English language publications (eg., Bardolph, 2014; Heath-Stout, 2020; Tushingham et al., 2017). There are numerous explanations for why men are still dominating archaeological scholarship, including that they are still hired at higher rates for research-intensive positions in the US and Canada and thus have fewer teaching responsibilities. Moreover, women are often employed in Cultural Resource Management or museum fields, which may not require publications for advancement, and men are less affected by demands associated with child and homecare. This may relate to, among other things, a chilly climate for women in academia and unexamined bias in hiring processes at major universities (for related discussions, see Fulkerson & Tushingham, 2019; Goldstein et al., 2018; Speakman et al., 2018).

All these explanations would account for the gender disparity in lidar publications given that lidar is expensive and male scholars are most likely to have stable positions and thus an institutional advantage when it comes to securing grant funds for such research. It is possible, however, that as the initial lidar data are more readily published, future papers will trend toward gender parity in publishing practices when early career scholars can more readily access extant datasets. Another factor is that women in many disciplines have suffered disproportionately in terms of academic research compared to their male counterparts during the first 2 years of the COVID-19 pandemic (2020–2021) (Caldarulo et al., 2022; Hoggarth et al., 2021; Malisch et al., 2020). Although longitudinal research will be necessary to fully document and account for the impacts of the pandemic on female-led scholarship, the emerging data suggest that school closures and stay-at-home-orders resulted in increased family care responsibilities for women and unequal distribution of domestic labor (Caldarulo et al., 2022). It would be interesting to explore this quantitatively within archaeological lidar literature in future.

Our exploration of authorship and local affiliation shows that publications are still dominated by individuals based at foreign (typically US and some European) institutions. This is apparent in overall authorship trends, but most salient in terms of primary authorship—there is only one paper first-authored by someone based in a Latin American country, and two papers led by people from Latin America but based at foreign institutions. Potential explanations for this include the high cost of lidar equipment acquisition and training in its use, its promotion at Latin American institutions, and English language publications being potentially not as important as Spanish or Portuguese papers, conference proceedings, government reports, etc.—in other words, what counts as authoritative work in parts of Latin America tends to be more locally than broadly defined. Regardless, authorship remains extremely unbalanced especially when we consider who is writing about geospatial data and pre-colonial landscapes in Latin America. Also unclear is whether first authors or local co-authors are affiliated with Indigenous or other stakeholder communities where scanning occurred. A survey of the authors included in this study would help to highlight this issue, but for now, it appears that Indigenous communities are even further away from having any kind of authoritative voice, and thus ownership of archaeological lidar data. Inclusion on academic publications would at least suggest collaboration, which is critical in archaeological research and particularly in pre-colonial/colonial landscapes such as in Latin America (Cohen et al., 2020; Palka et al., 2020; Sanger & Barnett, 2021).

Discussing efforts for increasing participation and long-term support are beyond our experience and therefore the scope of this paper; however, we want to explore a few ideas for how to enhance authorship of individuals with local affiliation and connections. First, it is important to acknowledge the wide range of variation throughout the Americas in terms of the development, governance, and inclusion of local and particularly Indigenous communities in academic research within their own spheres of influence. There are encouraging examples of academic research and data collection led and supported by Indigenous academics and community members in the US and Canada (eg., Cipolla et al., 2019; Colwell, 2016; Gonzalez, 2016; Marek-Martinez, 2021; Silliman, 2008); yet, the situation elsewhere is highly variable and the contexts of settler colonialism and indigeneity can be very region-specific and, therefore, not broadly comparable (González-Ruibal, 2019). For example, this variation is present within even small regions of Latin America. For instance, in Honduras, a country in which we conduct research, and which officially recognizes eight main Indigenous communities including Garifuna, the Miskitu (~ 13% of the overall Indigenous population) community has several members who hold significant political and academic authority within the county (Palacios, 2007; Insitituto Nacional de Estadistica, 2013). Smaller communities, such as the Tawahka (~ 0.5%), are still striving for greater representation and development. While there is a good track record of involvement of Miskitu individuals (who include academics) in research projects, including participatory mapping projects that supported their claims for land titles (Herlihy & Tappan, 2019), research projects that provide long-term sustainable support for continued capacity building and geospatial support are needed.

Similar to community collaboration in other forms of research, in lidar projects, such collaborations would have to be the result of a long, concerted, and sustainable effort toward more fully integrating the involvement of local communities. This would necessitate an understanding of local context, including the relationship between Indigenous peoples and government landowners, local place names, and micro-histories of colonialism and capitalism (Ardren, 2002; González-Ruibal, 2019; McAnany et al., 2015; Palka et al., 2020; Pyburn, 2011; Sletto, 2009). For some regions, this information may already exist and thus facilitate implementation of more integrated research strategies. For example, in the Maya Biosphere Reserve in the Péten, Guatemala, studies examine the socioeconomic performance of community enterprises (Stoian et al., 2018). This is potentially valuable for evaluating where lidar scanning would be useful for assessing issues of importance to those communities. It is also possible that archaeologists have already begun to incorporate some of this information into their research programs, but that this information is simply not made available in print generally, or in lidar-specific publications.

As a long-term goal, archaeological lidar projects should include some reflection on the practices that academics follow, from grant applications to data collection and ownership, to authorship. The CARE (Collective Benefit, Authority to Control, Responsibility, and Ethics) Principles, for example, would be a useful place to start for ideas on what Indigenous data sovereignty might look like across diverse regions of Latin America (Carroll et al., 2020). These principles discuss how data should facilitate collective benefit for Indigenous peoples and that Indigenous peoples should have the ability to access that data. Accountability and a concern for ethics that minimize harm are critical. While every region and lidar project area may be different, we should consider how we might work toward similar standards in archaeological lidar data collection.

## Conclusion

Scholars who work in Latin America, and Mesoamerica in particular, are increasingly using remote sensing technologies like lidar to document and better understand archaeological landscapes both in the past and today. Our study of lidar data ownership through publication authorship shows that male authors based at foreign institutions dominate scholarship. There does appear to be an increase in female first authors and co-authors, but it is uncertain when, or if, authorship will achieve gender balance. Authors who are affiliated with the countries in which scanning occurred are poorly represented, and it is unclear whether any authors have a community connection with the scanning locations. In the future, monitoring of these trends will be useful for assessing progress regarding ownership and diversity, including topics such as where lidar scans take place and whether some of the earlier publications on lidar datasets are accessible to scholars and communities where scanning has occurred, former graduate students, and early career scholars. Ultimately, our assessment of ALS in the archaeology of Latin America shows that there needs to be more discussion about collaborative research with Indigenous communities and stakeholders and the meaning of datasets and their impacts on Indigenous communities. Scholarship that points out how remote sensing data can help document diminishing landscapes and its effects on Indigenous communities today at risk from deforestation and modernization provide an important start for future projects.
